# Fatigue, sleep quality and mental health symptoms in Brazilian women during the COVID-19 pandemic: longitudinal study

**DOI:** 10.1038/s41598-022-23612-z

**Published:** 2022-11-27

**Authors:** Gabriel Bernardi dos Santos, Ana Carolina Sartorato Beleza, Tatiana de Oliveira Sato, Cristiano Carvalho, Paula Regina Mendes da Silva Serrão

**Affiliations:** 1 Rheumatology and Hand Rehabilitation Research Laboratory, Physical Therapy Department, Federal University of São Carlos (UFSCar), Rodovia Washington Luís, Km 235, Monjolinho, São Carlos, SP 13565-905 Brazil; 2 Women’s Health Research Laboratory, Physical Therapy Department, Federal University of São Carlos (UFSCar), Rodovia Washington Luís, Km 235, Monjolinho, São Carlos, SP 13565-905 Brazil; 3 Preventive Physiotherapy and Ergonomic Laboratory, Physical Therapy Department, Federal University of São Carlos (UFSCar), Rodovia Washington Luís, Km 235, Monjolinho, São Carlos, SP 13565-905 Brazil

**Keywords:** Psychology, Public health, Quality of life

## Abstract

To assess the impact of the COVID-19 pandemic on the variables of sleep quality, fatigue, anxiety, and depression in healthy Brazilian women. Longitudinal observational study conducted through an online questionnaire with women in 2020 and 2021. The Pittsburgh Sleep Quality Index, the Fatigue Severity Scale and the Hospital Anxiety and Depression Scale were used. The data were analyzed descriptively and the comparison between the data obtained in the first and second evaluation was performed using the McNemar test. A logistic regression was applied to test the association between the variables that showed a significant difference. A total of 235 women responded to the questionnaires. There was a significant increase in fatigue between the two moments (*p* < 0.05). In the first assessment, depression (OR: 2.39; 95% CI: 1.14–4.99), anxiety (OR: 2.68; 95% CI: 1.37–5.22) and sleep quality (OR: 4.01; 95% CI: 1.71–9.67) were associated with fatigue. In the second assessment, depression (OR: 2.93; 95% CI: 1.19–7.18) and anxiety (OR: 2.69; 95% CI: 1.27–5.71) were associated with fatigue. There was an impact on biopsychosocial aspects during the COVID-19 pandemic, with worsening of fatigue symptoms within a 6-month interval. In addition, fatigue was associated with symptoms of depression and anxiety, and worse sleep quality in the first year of the pandemic, remaining associated with symptoms of depression and anxiety in the second year of the pandemic in the country.

The COVID-19 disease, which emerged at the end of 2019, and the consequent pandemic declared by the World Health Organization in March 2020 ^1^ brought a series of social and health repercussions. At the end of 2020, the topic of mental health was at the top among the articles published on COVID-19 ^2^, which shows a high level of concern regarding this topic on the part of the global scientific community. Studies conducted in different countries found high rates of symptoms related to mental health such as anxiety (6.33% to 50.9%), depression (14.6% to 48.3%) reported in the general population, also, event impact scales in relation to stress, depression and anxiety had mean scores of up to 42.35 in Asia countries ^3,4^. A study carried out at the beginning of the pandemic evaluated the effects of social isolation on sleep quality, stress and anxiety and showed poor sleep quality and a high level of anxiety and stress in the population ^5^.

During the history of pandemics and epidemics, studies show that women’s health needs have never really been met, causing a high level of mental and physical distress ^6^. These needs are fundamental to be target, since depression, for example, is more common in women even before the COVID-19 pandemic ^7^. Studies demonstrated that fatigue and cognitive impairment such as depression are some of the sequelae of the COVID-19 infection ^8,9^. In addition, mental health was affected by the pandemic even in people who were not infected ^9^.

Additionally, it is known that women have a double workload, since in the family context, the care of children and/or the elderly and housework continue to be almost exclusively the responsibility of women ^10^. The challenge of balancing family, domestic, work, and social management increases the pressure on women’s time and energy and puts them at risk of great stress ^11^, emotional overload, which can harm their overall health and well-being ^12^. In the pandemic aspect, some factors may have led to an overload in the lives of these women, such as the closing of schools, teleworking, children at home, fear of getting sick, among other questions. In this context, more studies investigating the impact of the pandemic on aspects of mental health, fatigue and sleep quality in women are important.

Thus, the objective of this study was to evaluate the quality of sleep, fatigue and mental health during the first and second year of the COVID-19 pandemic in Brazilian women, performing a comparison between these two moments and verifying associations between the variables that demonstrate changes over time. Studies like this can generate measures to mobilize the world scientific community, leading to preparatory measures and necessary interventions, in addition to promoting the need for a better look at this population.

## Methods

The present longitudinal observational study was carried out through an online questionnaire, disseminated on the social networks and media of the University Federal of São Carlos. This study followed the recommendations of the CHERRIES (Checklist for Reporting Results of Internet E-Surveys) and was approved by the Ethics Committee for Research on Human Beings of University Federal of São Carlos nº CAAE 36469420.1.0000.5504. All methods were performed in accordance with the relevant guidelines and regulations by a statement provided by Ethics Committee for Research on Human Beings of University Federal of São Carlos and this study was performed in accordance with the Declaration of Helsinki.

The study was carried out between September 2020 (first assessment) and August 2021 (second assessment), first and second years of the pandemic, respectively. Inclusion criteria were: (1) women between 18 and 60 years of age; (2) not being diagnosed with any disease such as: fibromyalgia, osteoarthritis, systemic lupus erythematosus, hypertension, diabetes; (3) to be living in Brazil at both evaluation moments. Exclusion criteria were non-consent to participate in the research, male, not residing in the country during both assessments and not responding to both assessments. The final questionnaire used in the research is a composition of validated scales and a pilot test was carried out one month before the questionnaire was applied to students and women of different educational levels close to the researchers. The participants were the same during the two surveys, the authors ensured this by contacting them using the mandatory email address to access the platform of survey and in the second evaluation, it was necessary to access with the same email address. The study was widely publicized on social media and the local press.

The questionnaire was divided into five sessions, the first being about personal data (name, phone, email, diagnosis of COVID-19 or if someone close had the disease or died from the disease). The following sessions contained the questionnaires used to assess the outcomes: sleep quality (Pittsburgh Sleep Quality Index), fatigue (Fatigue Severity Scale) and anxiety and depression (Hospital Anxiety and Depression Scale) and the last session was composed of the contact of the main researcher and acknowledgments for participating in the research, totaling 5 pages. When accessing the online questionnaire, the participant had access to the Free and Informed Consent Form, the average time for completing the questionnaire and that their participation was voluntary, and that the data would be stored by the main researcher and used only for the benefit of the research, in addition to the main objectives of the study.

The questionnaire administration processes were performed by the first author of the study, the answers were collected and stored automatically through the Google Forms tool, and only the main researcher had access to the data. The platform used did not allow the participant to continue the questionnaire if any question was not answered and, at the end, she could check her answers.

### Pittsburgh sleep quality index

Sleep quality was assessed using the Pittsburgh Sleep Quality Index—IQSP (Pittsburgh Sleep Quality Index), which includes 21 items. This questionnaire was translated and adapted to Portuguese ^13^. The questions concern the quality of sleep during the last month. It has seven components: subjective sleep quality, sleep latency, sleep duration, sleep efficiency, sleep disturbances, use of sleep medications, and daytime dysfunction ^14^. For interpretation of results, a score ≤ 4 indicates good sleep quality and ≥ 5 indicates poor sleep quality.

### Fatigue severity scale

The Fatigue Severity Scale is a validated questionnaire translated into Portuguese ^15^, which relates the characteristics of fatigue to other diseases, with response options ranging from 1 to 7 points (disagree to agree), such as answer to nine questions related to how the patient felt during the previous week ^16^. The final score is the sum of all responses and values above 36 points suggest the presence of fatigue.

### Hospital depression and anxiety scale

The Hospital Depression and Anxiety Scale was developed as a screening tool for anxiety and depression in the general hospital setting but has also been shown to be valid in primary care and in the community ^17^. The scale is composed of 14 items and two subscales: 7 items that assess anxiety and 7 items that assess depressive state, with each item having four response options for the participant, generating a score of 0–3, thus, it is possible score 21 for anxiety and 21 for depression. The scale indicates that the result is evaluated as follows: scores 0–7 are considered normal values, 8–10 mild, between 11 and 14 moderate and 15–21 severe. One study proposes a cutoff score between 8–9 to determine anxiety and depression ^18^. This scale can assess the current state of the patient with an emphasis on the condition in the last few days. The scale was translated into Portuguese and validated in 2007 ^19^.

### Second evaluation

Through telephone or email contact (provided in the first evaluation), the volunteer was invited to answer the questionnaire again as soon as they completed 6 months after their first evaluation. If they agreed to participate again in the research, a new access link to the questionnaire was made available, which contained the same instruments used in the first evaluation. This evaluation made it possible to compare the outcomes evaluated six months after the first participation.

### Statistical analysis

Data were analyzed descriptively, by means of absolute and relative frequency, means and standard deviation. The comparison between the data obtained in the two assessments was performed using the McNemar test. From the outcomes in which there was a difference between the two moments, a logistic regression was performed to verify if there were associations between the outcome in question with age, race/ethnicity and the other outcomes (sleep quality, fatigue and mental health) performed through the stepwise model, so if the variable does not enter the model there are no estimators available. Analyzes were performed using the SPSS program (IBM SPSS Statistics for Windows, version 25.0. Armonk, NY, USA). The significance level adopted was 5%. There were no incomplete questionnaires to be analyzed, since the platform used only counted the questionnaires answered in full.

### Ethics approval and informed consent

The present study was approved by the Ethics Committee for Research on Human Beings of University Federal of São Carlos nº CAAE 36469420.1.0000.5504. All volunteers had to sign a free and informed consent for, prior to the evaluation.

### Consent for publication

All authors are aware of and authorize the publication of this manuscript.

## Results

The sample was composed by 235 women who answered the questionnaires at the two assessments. The response rate cannot be defined, since the study was divulgated on social media, and we cannot measure its reach. The attrition rate was 41%, since 400 women answered the questionnaires at the first and 235 at the second evaluation. Women who did not respond to the second evaluation due to the impossibility of contacting them through the e-mail or telephone provided in the first evaluation, were therefore excluded from the study. All answered the questionnaire, and the demographic and sample characterization results are shown in Table 1. The mean age was 29.5 ± 9.5 years, with most of the sample composed of white women (74.9%) and who live in the Southeast region (87.7%). Most of the women who participated in the study were single (68.1%) and had some type of paid activity (45.1%), followed by students or interns (44.7%).

**Table 1:** Sociodemographic characteristics of the women evaluated (n = 235).

Characteristics	N	%
Age, years [mean (SD)]	29.5	9.5
**Color/race**		
White	176	74.9
Black/Brown	49	20.8
Indigenous/Yellow	7	3.0
Do not wish to declare	3	1.3
**Region**		
North	3	1.3
Northeast	7	3.0
Midwest	5	2.1
Southeast	206	87.7
South	14	6.0
**Marital status**		
Single	160	68.1
Married	53	22.6
Divorced	12	5.1
Stable union	10	4.3
**Occupation**		
Student/intern	105	44.7
Unpaid activity	24	10.2
Paid activity	106	45.1

A difference was found in the proportions of women with and without COVID-19 between the two evaluated moments, as well as for women who reported the diagnosis of COVID in a close person and death from COVID in a close person, with the proportions of contamination and death increasing over time. over time (p < 0.01).

The comparison between the assessments of sleep quality, fatigue, depression and anxiety are presented in Table 2.

**Table 2:** Comparison between the data obtained in the two assessments.

Characteristics	2020		2021		*P*
	N	%	N	%	
Poor sleep or sleep disorder	199	84.7	187	79.6	0.10
Fatigue	163	69.4	189	80.4	< 0.01
Anxiety	152	64.7	157	66.8	0.58
Depression	100	42.6	102	43.4	0.90
Anxiety and depression	86	36.6	94	40.0	0.37

Regarding the fatigue index, there was a difference in the proportions of women with and without, between the two evaluated moments (X2(1) = 11.16; *p* < 0.01). In the first evaluation, the proportion of women with fatigue was 69.4% and after 6 months it increased to 80.4%.

There was no difference in the proportions of women with and without sleep disorders, symptoms of anxiety and depression, and also anxiety associated with depression, between the two moments evaluated. Regarding the questionnaire that evaluated aspects of mental health, anxiety symptoms were more frequent and at a high level also in the two evaluation moments, being 64.7% (152) and 66.8% (157) respectively. Depressive symptoms were reported by almost half of the sample, reaching a frequency of 43.4% in the second evaluation moment.

Based on the finding of a difference between the times for fatigue alone, with a significant worsening, an analysis of the association of this with age, color/race, sleep quality, mental health (isolated symptoms of depression and anxiety and the combination of both—depression and anxiety). The data are represented in Table 3.

**Table 3:** Association between fatigue and outcomes, sleep quality and mental health components.

Fatigue	First assessment			Second assessment		
	OR	95% CI	*p* value	OR	95% CI	*p* value
Depression	2.39	1.14–4.99	0.02	2.93	1.97–7.18	0.01
Anxiety	2.68	1.37–5.22	< 0.01	2.69	1.27–5.71	0.01
Poor sleep or sleep disorder	4.01	1.71–9.67	< 0.01	–	–	–

*OR* Odds ratio; *CI* confidence interval; There was no association between poor sleep or sleep disturbance in the second assessment.

In the first evaluation, fatigue was associated with symptoms of depression, anxiety, and sleep disorder/poor sleep, with depression increasing the chance of having fatigue by 2.93 times, anxiety increasing the chance by 2.68 times and poor sleep or sleep disorder by 4.01 times. In the second evaluation, the variables associated with fatigue were: symptoms of depression and anxiety, with an increase of 2.93 times and 2.69 times, respectively.

## Discussion

The present study aimed to evaluate the quality of sleep, fatigue and mental health during the first and second year of the COVID-19 pandemic in Brazilian women, comparing these two moments and also verifying associations between the variables that showed worsening. over time. Our findings indicated that women showed increased fatigue throughout the pandemic. Although we found no difference between the two collection times for aspects related to sleep quality, symptoms of anxiety and depression, and associated anxiety and depression, these aspects were impaired in this population, these results are similar with a previous study that found no differences in mental health variables across a longitudinal study ^20^.

Many women showed symptoms of depression and anxiety. The pandemic resulted in an increase in cases of depression and anxiety disorders worldwide, these data from the literature are reported by studies that investigates these aspects at the beginning of the pandemic, which may be a consequence of an acute reaction to the event pandemic ^21^, data that make it necessary to investigate these aspects over time. Although symptoms of mental health disorders in this context are expected to be followed by a state of resilience and improvement ^22^, our study showed an increase in depression and anxiety symptoms in women who participated in the study between the period evaluated. Also, our results agree with a study that evaluated the presence of anxiety and depression disorders in the general population, which found a high rate of these symptoms, especially in the population younger, in those who spent a lot of time thinking about the pandemic and in healthcare professionals ^23^.

Regarding the mental health of healthy women, data from the literature indicate that support for pregnant women during the pandemic is also necessary and there was an influence of COVID-19 on antenatal care, which shows another issue of vulnerability of this specific population ^24^. Also, a significant increase in cases of violence against women were reported in several countries ^25^, which may be related to social isolation and the difficulty of support ^6^. Studies show that domestic stress increases the likelihood of violence between partners and that social distancing, currently necessary, can be considered a risk factor for these women who live in situations of violence ^26,27^. In addition, it is possible to verify in the literature that the factors that increase women's vulnerability to violence were greater during the pandemic period ^28^. From these data, we can infer that the inequality imposed by the female gender and its aspects related to violence may have interfered with mental health issues in the pandemic.

Regarding fatigue data, it was observed that women showed an increase in fatigue over time, reaching 80.4% of the total sample. Fatigue symptoms and burnout were widely discussed during the pandemic, these symptoms can affect people’s ability to work or perform their daily life activities, this worrying and valid reality led to the creation of a specific scale to assess burnout and provide a contribution for the effective and early identification and monitoring, published July 2022 ^29^. The literature points out several factors that may be linked to the increase in fatigue in women during the pandemic, since, in general, daily activities related to the family and even leisure were affected, which caused women to be disproportionately affected, as this type of activity is usually attributed to females ^30^. In addition, there was also a decrease in the level of physical activity and an increase in daily sitting time, factors that may explain an increase in fatigue ^31^.

Although fatigue worsened between the moments evaluated by the study, there was no worsening in sleep quality, reaching a rate of 79.6% of the sample in the second year, lower than in the first year of the pandemic, where 84.7% of women had poor sleep or sleep disorder. Similarly, Li et al. (2022) evaluated the sleep quality in postpartum women in China, before and during the pandemic, and found that there was no worsening in sleep quality using the Pittsburgh Sleep Quality Index. They bring as possible hypotheses to this finding, such as the longer time with the family and Chinese government policies to guarantee food and medication to the population^32^. It is important to emphasize that, as in mental health, a high number of women had this problem, which increases the importance of evaluating and seeking methods to improve these variables. In addition, we highlight that in the first year of the pandemic, having a sleep disorder or poor sleep, anxiety or depression increased the chance of having fatigue. However, in the second year only anxiety and depression were associated with fatigue. This may be because even if there were sleep disorders, mental health aspects persisted in the population evaluated, impacting the fatigue of these women, although we cannot say that these aspects are due to the impact caused by the pandemic.

At the beginning of the pandemic, a study carried out in China showed that there was a high prevalence of poor sleep quality among the Chinese population during the COVID-19 outbreak ^23^. The literature shows consistent evidence regarding the use of cognitive behavioral therapy for insomnia in the online format, necessary during the pandemic, but with a low relationship to improving quality of life ^33^. In addition, a national study carried out in South Korea sought to find associations between some factors common to our work, identifying that, individually, fatigue and sleep disorders were highly associated with quality of life related to mental health in healthy individuals ^34^. These data corroborate our findings in relation to the associations found through logistic regression analysis, where fatigue was associated with sleep disturbance, but mainly with the variables of anxiety and depression in both evaluated moments, with an increase of 2.63 and 2.69 times of women with these symptoms have fatigue, which may explain the high levels of anxiety and depression found in the assessments and, consequently, a significant increase in fatigue between the evaluated moments.

This study has limitations that should be considered, for example, the response rate is unknown. The study did not investigate whether the study participants had a previous diagnosis of sleep disorders, such as apnea or insomnia, or the diagnosis of anxiety or depression, aspects that serve as a suggestion for future studies. It is important to emphasize that it cannot be said that such losses presented in the outcomes are linked to the pandemic, for this, an evaluation would be necessary before the beginning of the pandemic period, therefore, this study only evaluates the pandemic period and suggests that post-pandemic studies can investigate the prevalence of these aspects in the female population and use the present study as a comparative study. Future studies that may involve other variables and make associations with aspects such as pregnancy, number of children, history of domestic violence can help to develop a better view of the situation of Brazilian women during the pandemic.

The present findings show that symptoms linked to aspects of the quality of life of Brazilian women during the pandemic need to be considered and that intervention measures and health programs aimed at biopsychosocial support are extremely important in the country.

## Conclusions

We conclude that a high rate of women is experiencing an impact on biopsychosocial aspects in the period of the COVID-19 pandemic in Brazil over time, with some of them reporting worsening, such as fatigue symptoms, which had a high association mainly with symptoms of depression and anxiety.

## Data Availability

The authors declared that the data is available and the principal author, Gabriel Bernardi dos Santos, should be contacted if someone wants to request the data from this study.
